# Breathing and Singing: Objective Characterization of Breathing Patterns in Classical Singers

**DOI:** 10.1371/journal.pone.0155084

**Published:** 2016-05-09

**Authors:** Sauro Salomoni, Wolbert van den Hoorn, Paul Hodges

**Affiliations:** The University of Queensland, Centre for Clinical Research Excellence in Spinal Pain, Injury and Health, School of Health and Rehabilitation Sciences, Brisbane, Australia; Northwestern University, UNITED STATES

## Abstract

Singing involves distinct respiratory kinematics (i.e. movements of rib cage and abdomen) to quiet breathing because of different demands on the respiratory system. Professional classical singers often advocate for the advantages of an active control of the abdomen on singing performance. This is presumed to prevent shortening of the diaphragm, elevate the rib cage, and thus promote efficient generation of subglottal pressure during phonation. However, few studies have investigated these patterns quantitatively and inter-subject variability has hindered the identification of stereotypical patterns of respiratory kinematics. Here, seven professional classical singers and four untrained individuals were assessed during quiet breathing, and when singing both a standard song and a piece of choice. Several parameters were extracted from respiratory kinematics and airflow, and principal component analysis was used to identify typical patterns of respiratory kinematics. No group differences were observed during quiet breathing. During singing, both groups adapted to rhythmical constraints with decreased time of inspiration and increased peak airflow. In contrast to untrained individuals, classical singers used greater percentage of abdominal contribution to lung volume during singing and greater asynchrony between movements of rib cage and abdomen. Classical singers substantially altered the coordination of rib cage and abdomen during singing from that used for quiet breathing. Despite variations between participants, principal component analysis revealed consistent pre-phonatory inward movements of the abdominal wall during singing. This contrasted with untrained individuals, who demonstrated synchronous respiratory movements during all tasks. The inward abdominal movements observed in classical singers elevates intra-abdominal pressure and may increase the length and the pressure-generating capacity of rib cage expiratory muscles for potential improvements in voice quality.

## Introduction

Breathing patterns during speaking and singing can differ from that during quiet breathing by modification of respiratory kinematics (i.e. movements of rib cage and abdomen) in response to altered task demands. Active control of breathing pattern affects the efficiency of the respiratory system and is considered essential in classical singing training for the development of optimal voice performance [1–4]. Experienced singers and teachers commonly refer to the use of abdominal muscle “support” to improve respiratory control and tone quality [4–7]. Although an agreed definition of the term “support” remains elusive [8,9], it is generally considered to involve enhanced abdominal muscle activation, which elevates intra-abdominal pressure and expands the rib cage, thus increasing the length and the pressure-generating capacity of the rib cage expiratory muscles [10]. However, attempts to identify stereotypical patterns of respiratory kinematics in classically trained singers have been so far inconclusive [2,6,11,12], and it is unclear how the breathing pattern of classical singers differ from that of untrained individuals.

According to the National Association of Teachers of Singing, focus on abdominal breathing is one of the most effective directives when teaching breathing support [9,13]. Although the specific role of individual muscles during phonation remains debated [10,14–20], greater activation of abdominal muscles is generally observed during speaking and singing than quiet breathing [16,17]. From visual inspection of respiratory kinematics, it has been suggested that, during singing, classical singers contract abdominal muscles at the end of the inspiration phase, which is argued to produce pre-phonatory inward movement of the abdomen [2,12]. This would summate with the passive recoil characteristics of chest wall and lungs in preparation for efficient generation of expiratory airflow [4,21–23]. During phonation, contracted abdominal muscles prevent shortening of the diaphragm [17] and provides the opposing force required for the rib cage to develop strong subglottal pressure in order to increase sound pitch and/or loudness [2,6,15,24]. Furthermore, the elevated position of the ribs increases rib cage volume and allows for quick phonatory manoeuvres [25,26]. The independent and asynchronous movements between the rib cage and abdominal wall often results in paradoxical motion, characterized by compartmental volume displacement opposite in sign to lung volume change, such as increased volume of the rib cage during expiration/phonation phase of the breath cycle [2].

Previous studies have assessed classical singers during singing performances with and without use of the supported voice strategy [4,6,20,27]. The results suggest that the supported voice is associated with greater subglottal pressure, greater sound pressure, and higher peak airflow. Together, this leads to a requirement for larger air volumes to produce the same musical phrases and has been suggested to influence high frequency bands of the sound power spectrum [6,28]. However, two issues hinder the generalization of these findings. First, considerable inter-subject variability has been reported in most studies [6,29]. Second, although professional classical singers often repeat consistent patterns of respiratory kinematics when repeating the same musical task [30,31], when they are asked to perform with an “unsupported” voice, they must artificially emulate a non-habitual (and otherwise never used) breathing pattern, and it is unclear whether it is possible to avoid features of their own habitual patterns [7,32].

Studies that compare group averages of data recorded from classical singers with previous observations from untrained individuals [33] have suggested that professional singers initiate musical phrases at higher lung volumes [3], although conclusive statistical tests have not been performed. Moreover, classical singers showed greater deformation of the rib cage and abdominal wall in respiratory adjustments during singing than during speaking tasks [2,12], whereas untrained individuals and professional country singers used similar strategies during both tasks [33,34]. However, these observations have been based on qualitative/subjective assessments, often relying on visual inspection of raw data, thus providing limited support to infer about typical patterns of respiratory kinematics of classically trained singers. In order to identify the unique features of the breathing patterns of classical singers, it is necessary to perform direct and objective comparison between professional singers and untrained individuals.

The aim of the current study was to objectively characterize dynamic patterns of respiratory kinematics during quiet breathing and singing in order to: (i) compare the features of breathing patterns of professional classical singers with a group of untrained individuals during a standardised singing task; and (ii) investigate whether breathing patterns differed between quiet breathing and singing for each group. We hypothesized that, in contrast to untrained individuals, classically trained singers would demonstrate: (i) greater contribution of abdominal volume to total lung volume; (ii) weaker correlation between movements of the rib cage and the abdominal wall when singing; and (iii) this would reflect greater asynchrony between movements of the rib cage and abdomen during singing than quiet breathing. Finally, (iv) higher inter-subject variability is expected among professional singers than untrained controls due to the development of subject-specific techniques of respiratory kinematics.

## Methods

### Participants

Seven professional classical singers (two males, aged from 39 to 41; five females, aged from 24 to 72) and four untrained individuals (control group, all males, aged from 21 to 41) participated in this study. Table 1 presents demographic data and information about singing training and performing experience from participants (anthropometric information for classical singer #3 is not available). All participants were native English speakers, and provided written informed consent prior to inclusion. The Medical Research Ethics Committee of The University of Queensland approved the study, which was conducted in accordance with the Declaration of Helsinki.

**Table 1:** Anthropometric parameters and information about formal training and performing experience from classical singers and untrained individuals (controls).

	Classical							Control			
	#1	#2	#3	#4	#5	#6	#7	#1	#2	#3	#4
**Age (yrs)**	61	27	29	41	72	24	39	37	21	36	41
**Gender**	F	F	F	M	F	F	M	M	M	M	M
**Height (cm)**	165	160	N/A	178	165	172	182	184	175	173	182
**Weight (kg)**	60	61	N/A	75	56	82	86	84	65	64	69
**BMI (kg/m2)**	22.0	23.8	N/A	23.6	20.5	27.7	25.9	24.8	21.2	21.3	20.8
**Training experience (yrs)**	7	5	10	8	20+	6	10	-	-	-	-
**Performing experience (yrs)**	35	8	10	21	30+	6	20	-	-	-	-
**Full- or Part-time Singer**	Part-time	Part-time	Full-time	Full-time	Full-time *	Full-time	Full-time	-	-	-	-
**Solo or Choral Singer**	Both	Choral	Solo	Solo	Both	Solo	Solo	-	-	-	-
**Recital or Opera Singer**	Recital	-	Opera	Opera	Both	Both	Opera	-	-	-	-

Notes: Anthropometric parameters not available (N/A) for classical singer #3.

* Classical singer #5 currently works primarily as a singing teacher.

F: Female.

M: Male.

### Inductance plethysmography

Respiratory kinematics were estimated using respiratory inductance plethysmography bands (Inductotrace, Ambulatory Monitoring Inc., USA). One band was placed around the rib cage with the upper edge below the axilla, and the other placed around the abdomen, between the lowest rib and the iliac crest. In order to minimize potential effects of temperature-related baseline drift in the signal, a warm up period of 15–30 minutes was allowed after placing the elastic bands. Moreover, volume parameters were calculated within individual breath cycles, during which the baseline drift was negligible.

### Airflow

Airflow was recorded using a pneumotachograph (Hans Rudolf Inc., USA) connected to a differential pressure transducer (Validyne Engineering, USA). Phonation was recorded using a headset microphone (Allans Billy Hyde, Australia), and was used to assist identification of event timings in the respiratory data. All analogue signals were A/D converted and recorded at 2 kHz using a CED1401 data acquisition system and Spike2 software (Cambridge Electronic Design, UK).

### Experimental procedure

Data acquisition started with the calibration of the pneumotachograph using a 3000 mL precision syringe (Hans Rudolph Inc., USA). Participants then performed an isovolume manoeuvre, which consisted of alternate expansion of the rib cage and abdomen with the glottis closed, for estimation of the total lung volume as the weighted sum of rib cage and abdominal compartmental volumes [35]. Following the calibration procedures, each participant performed the following tasks in standing position:

1. Quiet breathing for 1 minute;
2. Singing the traditional Australian song *Waltzing Matilda*, which was well known to all participants. Classical singers sang this song in operatic style, whereas control participants sang in the traditional folk style;
3. Singing a piece of the participant’s choice (1–2 minutes), which for the classical singers involved an operatic piece. This task was chosen to observe respiratory kinematics when subjects performed a song with which they were familiar and allowed classical singers to demonstrate their usual singing technique.

Tasks 2 and 3 were repeated twice, and were performed without instrumental accompaniment (i.e. “a capella”). The first repetition was completed without a facemask, whereas in the second participants sang wearing a pneumotachograph facemask over the mouth and nose. An experimenter stood next to the participant to support the weight of the facemask, such that participants could maintain the same body posture during both repetitions without additional load to the head and neck, which could have influenced their breathing patterns.

### Data analysis

Rib cage (RC) and abdominal wall (AB) plethysmographic signals were calibrated using the weightings derived from the isovolume manoeuvre and the volume changes measured with the pneumotachograph. Total lung volume was estimated as the weighted summation of RC and AB signals. Data were low-pass filtered at 10 Hz (second-order Butterworth filter) to remove high-frequency instrumentation noise. Individual breath cycles (inspiration and expiration phases) were identified from local maxima and minima lung volume, with reference to the airflow (when available) and audio signals to aid the identification process. Inspiration phase was identified as the periods of negative airflow, with a transition from minimal to maximal lung volume. Similarly, expiration/phonation phase was identified as the period from maximal to minimal lung volume, with positive airflow and activity of the audio signal. Although some studies have identified phonation phase solely based on audio events [31], this procedure ignores the transition between inspiration and expiration/phonation phases, which is of particular interest in the characterization of breathing patterns.

**Fig 1:**
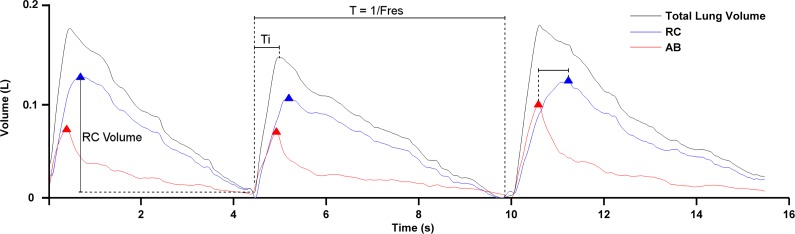
Illustration of rib cage (RC), abdominal (AB) and total lung volumes during three breath cycles of a classical singer performing a singing task (task 1: Waltzing Matilda). Parameters assessed from respiratory waveforms are indicated: respiratory frequency (Fres), time of inspiration (Ti), RC volume and time shift between peak volume of RC and AB.

The following parameters were extracted from the RC and AB volume signals and averaged over all breath cycles for each participant: Respiratory frequency (Fres, breaths/minute), time of inspiration (Ti, seconds), and percentage of rib cage contribution to total lung volume (%RC) (Fig 1).

The association between movements of the RC and AB was assessed using different parameters. The similarity between RC and AB was measured using the Pearson coefficient of linear correlation (*r*), averaged across all individual breath cycles. The amount of asynchrony between volume compartments was estimated by maximal cross-correlation [1] and expressed as phase angle. In order to investigate the transition between inspiratory and expiratory phases, the time shift between the moments of maximal volume of RC and AB was estimated (Fig 1). Consistent with the interpretation of the phase angle, a positive time shift was defined as a peak in AB volume occurring before that in RC volume. The duration of paradoxical motion of each volume compartment during expiration/phonation phase (i.e. paradoxical expansion of the RC or AB, reflecting asynchrony in respiratory kinematics) was quantified during each breath cycle and expressed as percentage of breath cycle length. The corresponding air volume moved during the period of paradoxical motion was expressed as percentage of total expiratory volume.

The airflow signal from the pneumotachograph was calibrated against the high-precision syringe using a third order polynomial fit procedure [5], and corrected to account for the difference in dynamic viscosity between inspired and expired gases in BTPS (body temperature and pressure, saturated with water vapour) and ambient conditions. The corresponding respiratory volume was estimated by numerical integration of the differential airflow signal. The calibrated airflow and volume signals were then used to assess peak airflow, mean airflow, and volume excursion during expiration/phonation phase (i.e., the difference between the volume at phase initiation and termination). Airflow data was not recorded for one classical singer for technical reasons.

**Fig 2:**
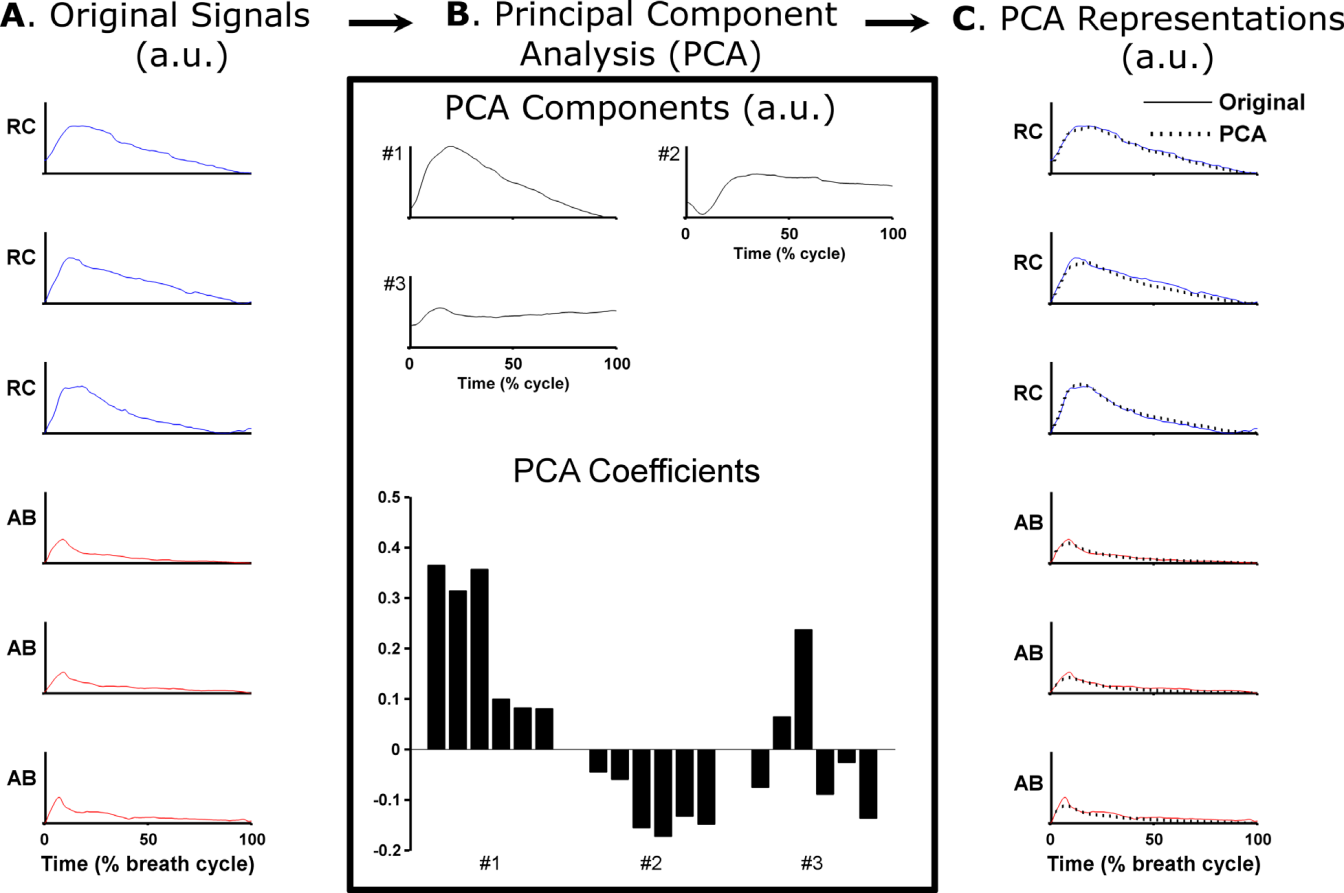
Example of extraction of typical breathing patterns using Principal Component Analysis (PCA). In A, rib cage (RC) and abdominal (AB) volumes are shown for three consecutive breath cycles of a classical singer performing a singing task (task 1: Waltzing Matilda). These waveforms were used to calculate the PCA components and PCA coefficients shown in B. In this example, three components explained more than 98% of the total variability of the original signals. The PCA representations in C were obtained by weighting the PCA components by the corresponding PCA coefficients. The typical breathing pattern for this subject (not shown) was obtained by weighting the PCA components by the average PCA coefficients across all breath cycles. a.u.: Arbitrary units.

Finally, in order to objectively characterize the typical breathing patterns of classical singers and untrained individuals, Principal Component Analysis (PCA) was performed on RC and AB volume signals (Fig 2A). Briefly, PCA is a mathematical procedure used to identify common patterns from large sets of input signals [8]. In the current study, the volume signals from all breath cycles were used as input signals for the PCA to generate a reduced new set of signals, called Principal Components, reflecting the most common features of the input data set. Each input signal was then represented as a weighted sum of a small number of Principal Components, where the weights are called PCA coefficients (Fig 2B). Usually in human movement analysis, the first Principal Component alone is able to explain more than 50% of the variability of all input signals, and the inclusion of additional Components gradually improves the accuracy of the PCA representation [11]. In the current study, the number of Principal Components was determined using a scree test, retaining all components with eigenvalues greater than 0.5% of the total variance (Fig 2C). As the length of the breath cycles were not identical, volume signals were time-normalized to 1,000 data points and expressed as a percentage of cycle length [13]. The amplitude of RC and AB volume signals was normalized in relation to the maximal lung volume of the corresponding breath cycle, therefore retaining the relative contribution of each compartment. Moreover, this method of amplitude normalization also compensates for differences in lung volume across multiple breath cycles. Classical singers and untrained individuals were assessed independently in order to identify the typical breathing patterns of each participant group [36]. The input for the PCA included RC and AB volume signals from all breath cycles of each participant, and the average PCA coefficients were multiplied by the Principal Components to obtain the participant’s breathing pattern. To derive the typical breathing pattern of each group, an additional analysis was performed including RC and AB signals from all subjects within that group. The same procedure was repeated for each experimental task. Inter-subject variability in the PCA representations of RC and AB signals of each group was assessed using the Variance Ratio (VR), which takes into account time and amplitude features of the waveforms [37].

$$VR=\frac{\sum_{i=1}^{k}\sum_{j=1}^{s}{\left(X_{ij}-{\bar{X}}_{i}\right)}^{2}/k\left(s-1\right)}{\sum_{i=1}^{k}\sum_{j=1}^{s}{\left(X_{ij}-\bar{X}\right)}^{2}/\left(ks-1\right)}$$

where *k* is the number of samples in the PCA representation (i.e. 1,000); *s* is the number of participants in the corresponding group; *X*_*ij*_ is the value at the *i*th sample for the *j*th participant; ${\bar{X}}_{i}$ is the average value of the *i*th sample over all subjects, and $\bar{X}$ is the average over all samples.

### Statistical analysis

Repeatability of parameters in conditions with and without facemask was evaluated using intra-class correlation coefficients (ICC). A one-way analysis of variance (ANOVA) was performed to compare the anthropometric data between groups (classical, control). Paired t-tests were used to assess the effect of facemask over repetitions of tasks 2 and 3 for each parameter. As no significant differences were found (all: p > 0.20), data from repetitions with and without facemask were pooled before further analysis, including PCA. In order to investigate group differences during each task, a 1-way ANOVA was applied, using group (classical, control) as between-subject factor, to each dependent variable: respiratory frequency (Fres), time of inspiration (Ti), percentage of rib cage contribution to total lung volume (%RC), phase angle and time shift between RC and AB, correlation coefficient between RC and AB, percentage of time and volume in paradoxical motion of RC and AB, peak and mean airflow, and total volume excursion. An additional 2-way repeated measures ANOVA was performed on the same dependent variables in order to assess changes between breathing and singing, with task (quiet breathing, *Waltzing Matilda*, own piece) as within-subject factor and group (classical, control) as between-subject factor. Duncan’s post-hoc test was used where appropriate with correction for multiple comparisons. Results are reported as mean ± standard error of the mean (SEM).

## Results

**Fig 3:**
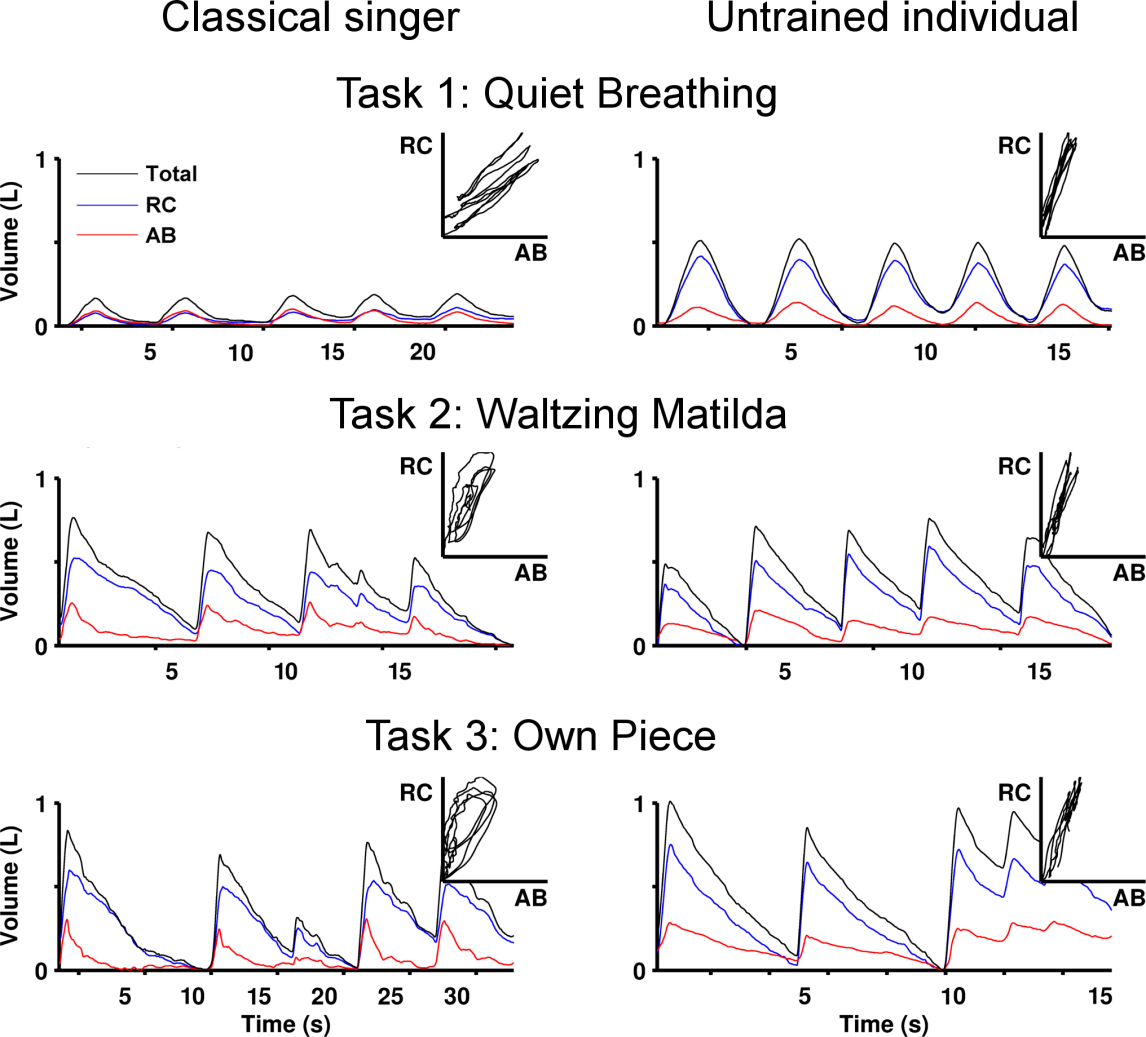
Representative recordings of rib cage (RC), abdominal (AB) and total lung volumes of one classical singer and one untrained individual performing each experimental task. Volume waveforms are shown for five consecutive breath cycles, with the corresponding Konno-Mead plots representing the coordination between AB and RC movements (in the x- and y-axis respectively).

**Fig 4:**
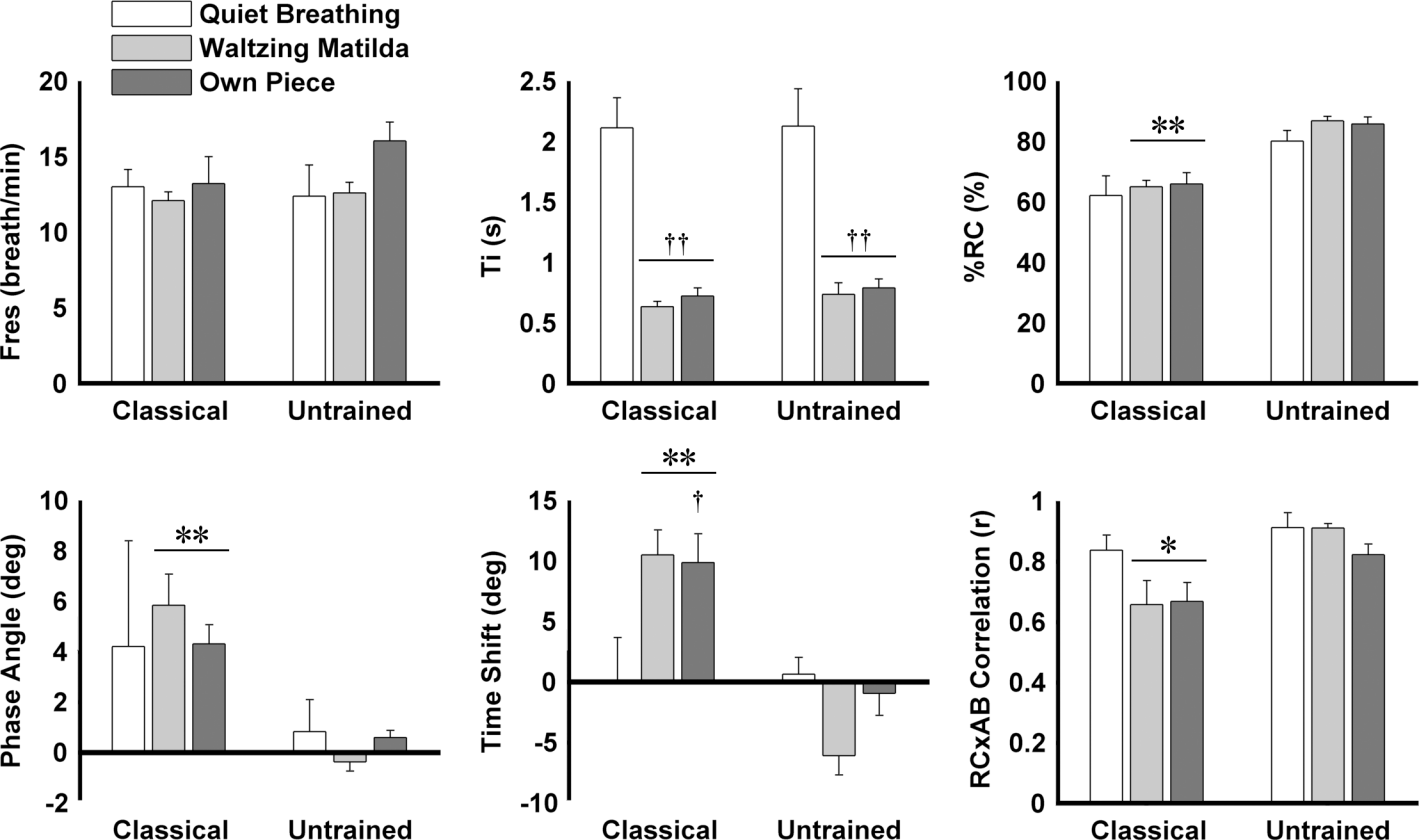
Respiratory frequency (Fres), time of inspiration (Ti), percentage contribution of rib cage to total lung volume (%RC), phase angle, time shift, and linear correlation coefficient between rib cage (RC) and abdomen (AB) volume waveforms of classical singers and untrained individuals during each of the three tasks assessed. Mean + SEM are shown. * P < 0.05, ** P < 0.001 vs. untrained individuals. † P < 0.05, †† P < 0.001 vs. quiet breathing.

**Fig 5:**
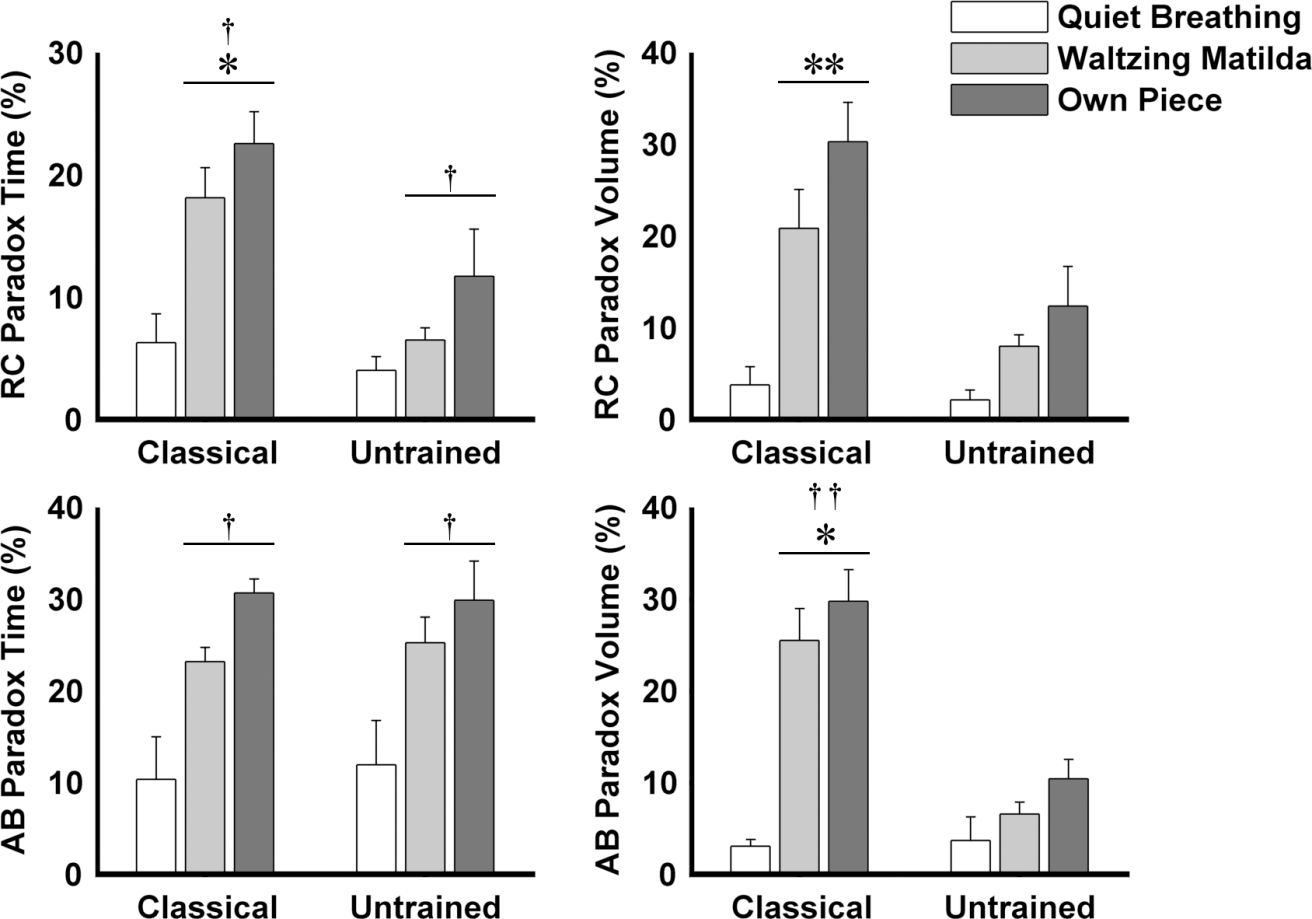
Percentage of time and volume in paradoxical motion of rib cage (RC) and abdomen (AB) of classical singers and untrained individuals during each of the three tasks assessed. Paradoxical motion is defined here as outward movements of the volume compartment during the expiratory phase of each breath cycle. Mean + SEM are shown. * P < 0.05, ** P < 0.001 vs. untrained individuals. † P < 0.05, †† P < 0.001 vs. quiet breathing.

**Fig 6:**
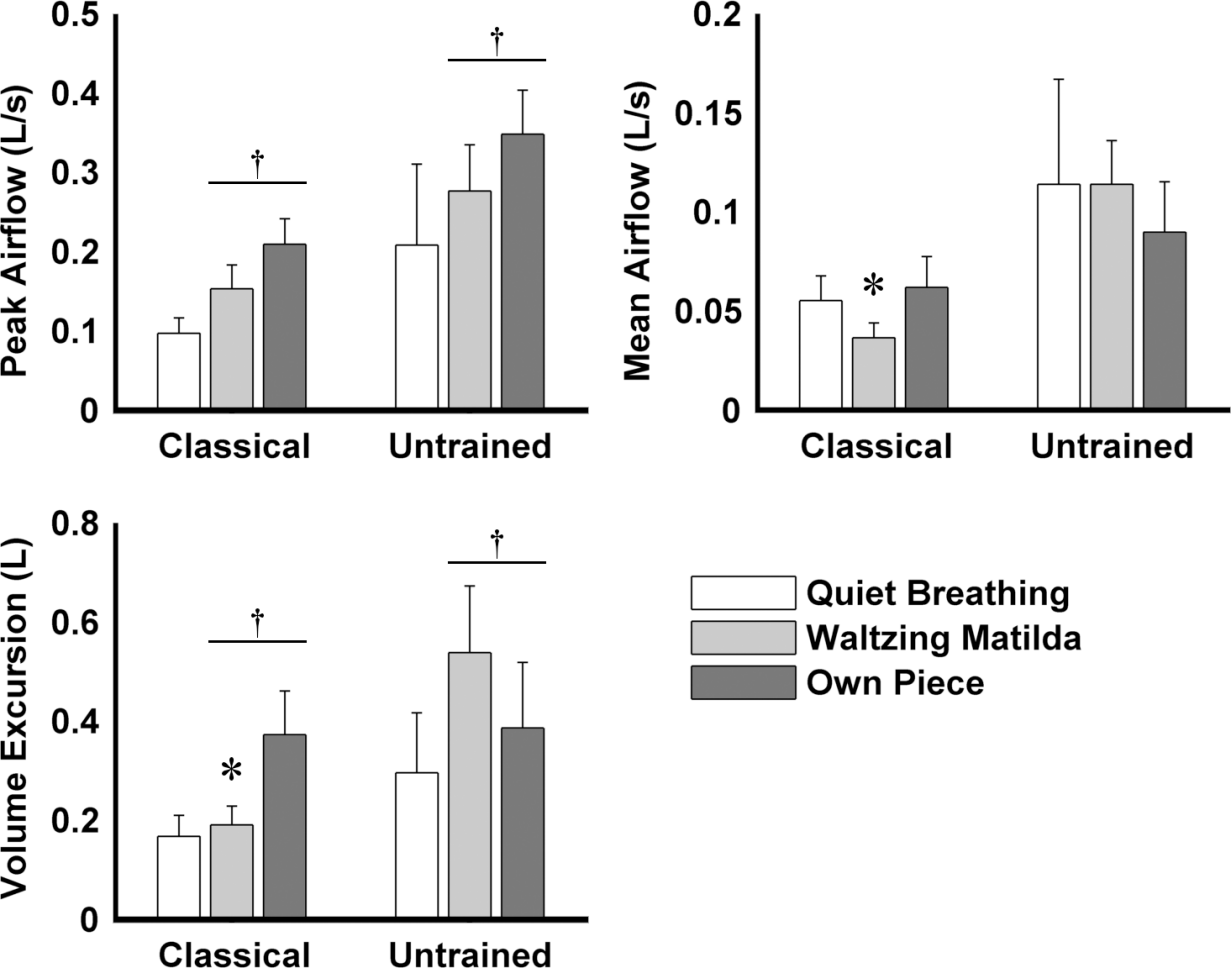
Peak airflow, mean airflow, and volume excursion of classical singers and untrained individuals during each of the three tasks assessed. Volume excursion was assessed as the difference between the volume at initiation and termination of expiratory phase of each breath cycle. Mean + SEM are shown. * P < 0.05 vs. untrained individuals. † P < 0.05 vs. quiet breathing.

Acceptable repeatability was observed in all parameters assessed for tasks performed with and without the facemask (ICC > 0.75). This supports the absence of an effect of the facemask on breathing strategy and consistency of the measures between separate trials. No differences were found in the demographic data between groups (see Table 1, 1-way ANOVA–no effect for group: Classical vs. Control Mean(SD); age 41.9(18.2) vs. 33.7(8.8) years, F(1,9) = 0.68, p = 0.43; height 170.3(8.5) vs. 178.5(5.3) cm, F(1,8) = 2.87, p = 0.13; weight 70.0(12.7) vs. 70.5(9.3) kg, F(1,8) = 0.00, p = 0.95; BMI 23.9(2.4) vs. 22.0(1.6), F(1,8) = 1.59, p = 0.24). Fig 3 shows representative examples of raw data recorded from a typical participant in each group, together with the Konno-Mead plots, which represent the coordination between AB and RC movements in the x- and y-axis, respectively. Although there were no significant differences between groups for any parameter during quiet breathing (1-way ANOVA–no effect for group: all F(1,9) < 3.98, p > 0.08), breathing patterns during singing differed in several key aspects. During the two singing tasks, classical singers used a smaller percentage of RC contribution to lung volume than untrained individuals (Fig 4, F(1,9) > 28.87, p < 0.001). Classical singers also demonstrated greater positive phase angle and time shift between RC and AB (i.e. AB before RC, F(1,9) > 28.92, p < 0.001), as well as greater percentage of time and volume of RC in paradoxical motion (i.e. motion of RC in direction opposite to that expected for direction of airflow, Fig 5, F(1,9) > 7.6, p < 0.05) and greater percentage of AB volume in paradoxical motion (F(1,9) > 10.93, p < 0.01). As a consequence of these features, smaller correlation coefficients were found between RC and AB signals of classical singers than controls during singing (F(1,9) > 5.7, p < 0.05). During the singing of the standard piece (*Waltzing Matilda*), lower mean airflow and lower volume excursion were observed in classical singers than controls (Fig 6, F(1,8) > 8.9, p < 0.05), but there was no effect of group for these parameters when participants sang the piece of their choice.

For both participant groups, in contrast to quiet breathing, singing tasks were associated with shorter duration of inspiration (Fig 4, 2-way ANOVA–main effect for Task: F(2,18) = 51.22, p < 0.001; post hoc: p < 0.001), greater percentage of time of RC and AB paradoxical motion (Fig 5, 2-way ANOVA–main effect for Task: F(2,18) > 22.44, p < 0.005; post hoc: p < 0.01), greater peak airflow, and greater volume excursion (Fig 6, 2-way ANOVA–main effect for Task: F(2,16) > 7.88, p < 0.005; post hoc: p < 0.05). The time shift between RC and AB of classical singers was greater when singing their own piece than during quiet breathing (2-way ANOVA–interaction Task × Group: F(2,18) = 4.43, p = 0.05; post hoc: p < 0.05), whereas no differences were found for untrained individuals (post hoc: p > 0.35). AB volume in paradoxical motion in classical singers was greater during both singing tasks than during quiet breathing (2-way ANOVA–interaction Task × Group: F(2,18) = 9.18, p = 0.02; post hoc: p < 0.001), but not in untrained individuals (post hoc: p > 0.13).

**Fig 7:**
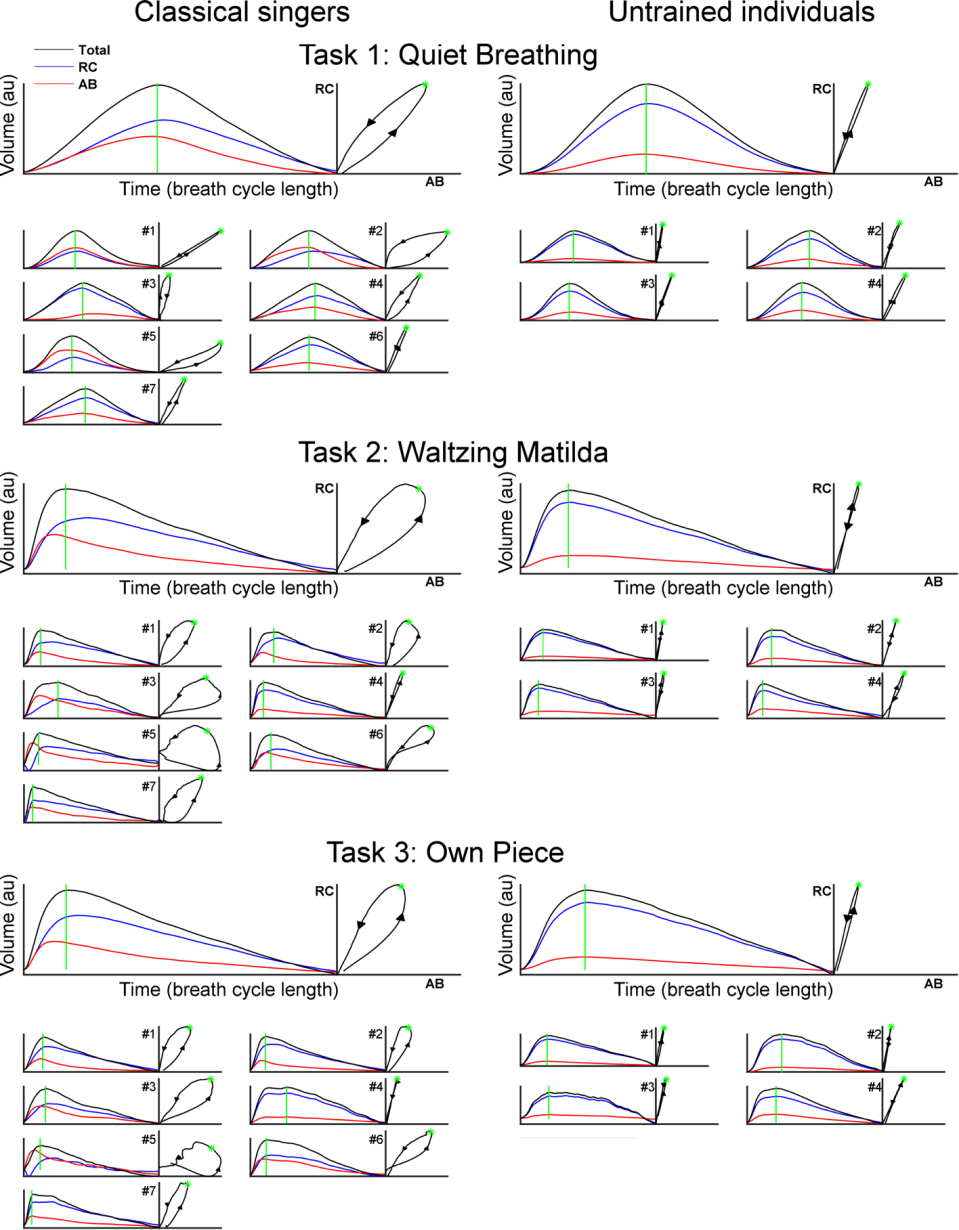
Typical breathing patterns of classical singers and untrained individuals extracted using principal component analysis. The group’s typical patterns are represented in the large axes, whereas patterns of individual participants are represented in small axes. Volume waveforms are shown with the corresponding Konno-Mead plots of RC and AB movements. The arrowheads in the Konno-Mead plots illustrate the time course, i.e. ascending and descending arrows correspond to inspiratory and expiratory phases, respectively. The start of expiration phase is marked in green both in the time plots and Konno-Mead plots. Note: The raw data shown in Fig 3 were recorded from classical singer #1 and untrained individual #4.

The patterns of RC and AB movements, estimated using principal component analysis (PCA), are depicted in Fig 7. The waveforms obtained account for more than 98% of the variability of the original signals and therefore represent the typical patterns of each participant group, as well as the patterns of individual subjects. For both groups, more Principal Components were required to represent RC and AB volume signals during singing than breathing (Table 2, 2-way ANOVA–main effect for Task: F(2,18) = 17.65, p < 0.001; post hoc: p < 0.001). On average, the patterns of classical singers required a larger number of Principal Components than untrained individuals, but the group difference narrowly missed statistical significance (Table 2, 2-way ANOVA–main effect for Group: F(1,9) = 4.74, p = 0.057). The graphical presentation of PCA data shows high inter-subject variability of breathing patterns between classical singers, whereas similar patterns were observed within the group of untrained individuals. As a result, greater Variance Ratio was observed for classical singers than controls (Table 2). Furthermore, classical singers showed strong asymmetry in the coordination between RC and AB movements, as demonstrated by “open” loops in the Konno-Mead plots, which were generally wider during singing tasks than quiet breathing. Untrained individuals, on the other hand, repeated similar closed loops in the Konno-Mead plots during quiet breathing and singing tasks, despite differences in the time of inspiratory and expiratory phases between tasks.

**Table 2:** Number of Principal Components (PC, mean ± SD) and Variance Ratio (VR) of PCA representations of RC and AB volume signals for classical singers and untrained (control) individuals.

Parameter	Quiet Breathing		Waltzing Matilda		Own Piece	
	Classical	Control	Classical	Control	Classical	Control
Number of PCs	2.86 ± 1.07	2.25 ± 0.5	5.43 ± 1.62	3.75 ± 0.5	5.86 ± 1.57	4.75 ± 0.5
VR RC	0.444	0.056	0.231	0.049	0.303	0.131
VR AB	0.420	0.299	0.324	0.218	0.362	0.440

Note: PCA: Principal Component Analysis. RC: Rib cage. AB: Abdomen.

This study performed an objective characterization of the breathing patterns of classical singers and untrained individuals by means of Principal Component Analysis, in addition to a number of features of respiratory kinematics. Results show no significant differences between groups during quiet breathing, although the presence of small differences cannot be completely excluded because of the small sample size. In particular, the data suggest a trend for greater abdominal contribution in singers than untrained individuals during quiet breathing (1-way ANOVA p = 0.08), illustrated in Figs 4 and 7. During singing tasks, classical singers used consistently greater percentage contribution of abdomen to total lung volume and moved the abdomen inward prior to phonation, which resulted in higher asynchrony between movements of the rib cage and the abdominal wall than untrained individuals. As a result, “open” loops were observed in the Konno-Mead plots of the PCA representation of classical singers, i.e. distinct patterns of interaction between RC and AB during inspiration and expiration. These data highlight strong manipulation (most likely voluntary) of the patterns of respiratory kinematics by classical singers during singing tasks from the patterns naturally adopted during quiet breathing. This argument is supported by the observation of greater phase shift between RC and AB, greater contribution of AB to total lung volume, and greater inter-subject variability among classical singers than untrained individuals. Taken together, these data suggests the development of individual-specific respiratory techniques in classical singers, whereas untrained individuals consistently repeated coordinated movements of RC and AB during breathing and singing.

### Breathing patterns during singing

The absence of group differences in respiratory frequency and duration of inspiration during singing suggests that both groups adapted similarly to time constraints imposed by musical rhythm. In addition, both groups showed predominance of RC contribution to total lung volume, which might be interpreted as greater contribution of RC muscles over AB muscles to generate volume change. This would be consistent with observations that: (i) the RC covers a larger surface of the lungs, which enables small RC movements to produce relatively large changes in alveolar pressure; and (ii) the small RC expiratory muscles, such as m. intercostales interni and m. triangularis sterni, can provide fast and precise control of subglottal pressure, particularly when lung volume is below functional residual capacity [10,14,17,19]. Despite the predominance of RC motion, and consistent with the emphasis placed on abdominal breathing in operatic singing training [9], classical singers used larger contributions of AB, i.e. smaller %RC, than untrained individuals. On average, classical singers used 35% contribution of the abdomen during singing tasks, approximately 2.5 times the average contribution used by untrained individuals (14%). This corroborates previous reports based on subjective interpretation of raw data, which suggested greater deformations of the abdominal wall of classical singers than untrained individuals during singing [2,33].

Despite the short time of inspiration (mean 0.7 s), classical singers used inhalation manoeuvres that involved complex movements of respiratory compartments: In most singers, the AB wall began to expand prior to RC (i.e. positive phase angle), and AB peak volume occurred earlier than RC peak volume at the transition from inhalation to phonation (i.e. positive time shift). As a result, inward movement of AB started before maximum lung volume was reached, as depicted by the raw data in Fig 3 and the PCA representations in Fig 7. Such volume shift from AB to RC has been previously observed in classical singers in the transition from inspiration to phonation phase [2]. One interpretation of this early movement of the abdominal wall is that it can aid inspiration by stabilisation of the central tendon (control of the descent of the diaphragm) to enable the diaphragm to generate greater effect on the rib cage, i.e. rib elevation and lateral expansion [38]. This would be particularly important in standing position, where the gravitational effect on the abdominal viscera tends to lower the abdominal contents and thus the diaphragm position, shortening its muscle fibres. Moreover, the inward displacement of AB moves the diaphragm towards the thorax, which improves its position in the length-tension curve for the production of rapid changes in airflow, as a lengthened diaphragm yields more effective translation of muscle tension into transdiaphragmatic pressure [2,39–41]. Furthermore, as a result of the expansion of RC, pre-phonatory transfer of lung volume from AB to RC generates greater respiratory recoil forces and increases contractile force of expiratory rib cage muscles [4,42]. During phonation, contraction of abdominal muscles would elevate intra-abdominal pressure [43,44] and thus allow more efficient control of subglottal pressure required for sound production. Supporting this argument, greater activation of the obliquus internus and externus abdominis, and rectus abdominis muscles has been observed in professional operatic singers than students during singing tasks [24]. This has been related to changes in peaks of the sound power spectrum (known as formant frequencies) that are considered reflective of greater classical voice quality [6,28,45].

In the current study, strong agreement was observed in respiratory kinematics between singing tasks performed with and without a facemask. This demonstrates reliability of the experimental set up and repeatability of the breathing strategies used by both groups [30,31,46]. It should be noted that intra-subject repeatability of breathing patterns was not limited to rhythm-related constraints to the duration of inspiration and phonation phases that has been previously reported [23,46], but was also observed for the dynamics of respiratory kinematics (e.g. phase angle between RC and AB). This repeatability most likely reflects consistency of personal singing skills and respiratory strategies.

On the other hand, high inter-subject variability of respiratory kinematics has been commonly reported among classical singers [24,29]. During performances of the vocal exercise known as *messa di voce*, in which the loudness of a note is gradually increased and then decreased without changing other voice features such as pitch or timbre, singers with long performing experience demonstrated individual-specific patterns of respiratory kinematics, as opposed to the more stereotypic behaviour of less trained singers [47]. The breathing patterns shown in Fig 7 demonstrate similar observations during quiet breathing and the performance of full songs (i.e. *Waltzing Matilda* and the participant’s own piece of choice). Although the breathing patterns were similar among all untrained individuals, professional classical singers showed high degree of heterogeneity. This resulted in greater Variance Ratio and individual-specific breathing dynamics, as clearly shown by the Kono-Mead plots (Fig 7). As an example, singer #5 was the only one to demonstrate inward movements of RC at the start of inspiration, despite previous suggestion that this is a common behaviour among classical singers [4]. Moreover, singer #4 displayed a pattern similar to that of untrained individuals, despite having more than 21 years of performing experience. Taken together, these data highlight that although it is presumed that breath management techniques may provide physiological and mechanical benefits that optimise classical voice quality (through active modification of respiratory kinematics from the patterns spontaneously adopted by untrained individuals), these are not uniformly used by professional singers. This may imply that voice quality can be optimised by a range of mechanisms in addition to these standard techniques, e.g. dynamic changes in laryngeal and glottal configuration. This would partially explain the limited correlation between acoustic parameters and respiratory movements reported in previous studies [4].

### Breathing and singing

In addition to time constraints related to musical rhythm, singing tasks require more rapid inspiration and longer expiration for sustained utterances than quiet breathing, and this often results in end-expiratory volumes below functional residual capacity [48]. Accordingly, the current results indicate that classically trained and untrained singers adjusted the breathing patterns to singing task demands by decreased duration of inspiration, greater peak airflow, and greater volume excursion than during quiet breathing. However, changes in the time shift between RC and AB were observed only in classical singers. This suggests untrained individuals used similar strategies of respiratory movements during quiet breathing and singing tasks. Assessment of PCA representations demonstrates that, despite substantial differences between the time profiles of respiratory kinematics between tasks, the coordination between RC and AB of untrained individuals remained virtually unchanged. This observation agrees with and extends the findings of (i) significant correlation between the slopes of the Konno-Mead plots of RC and AB movements of untrained individuals during quiet breathing and reading tasks [49]; and (ii) similar Konno-Mead plots (based on visual inspection of raw data) when untrained individuals performed speaking, reading and singing tasks [33].

In contrast to untrained individuals, classical singers demonstrated greater time shift between RC and AB during singing than quiet breathing, and this was associated with greater AB volume in paradoxical motion. These findings suggest independent coordination of RC and AB muscles in classical singers, particularly in the transition from inspiration to phonation. Early inward movements of AB increase intra-abdominal pressure and diaphragm elevation prior to phonation [2]. This enables quick airway modulation during utterances and increases glottal efficiency, i.e. greater ratio between acoustic and aerodynamic power [6,50]. Moreover, classical singers often use most of their inspiratory vital capacity when singing [2], generating stronger recoil forces than untrained individuals, who are usually limited to volumes around the mid-range of vital capacity [33]. This enhanced efficiency could explain the current observation of lower mean airflow used by classical singers than untrained individuals during the performance of a similar singing task (i.e., *Waltzing Matilda*), as well as previous reports of lower subglottal pressure in classical singers than both country and musical theatre singers during speaking and singing tasks [51,52]. In line with this argument, classical singers can increase sound pressure level without changes in respiratory effort when singing with supported compared with unsupported voice [6]. In that study, the increase was attributed to changes in laryngeal and glottal configuration, such as lowering the larynx and tight closing the glottis, which was also associated with clearer voice quality. Despite this difference during the standard song, when required to perform at high vocal intensity, classical singers can develop greater airflow than non-singers. With a more efficient use of the vocal tract, this results in greater sound pressure level for the same lung pressures [53,54]. Although the current results do not imply the existence of an “optimal” method of training the singing voice, it underpins the importance of breath kinematics for classical singers, particularly the independent control of RC and AB movements.

One issue to consider when assessing breathing patterns in different individuals is that body type affects breathing and speech [55], and previous studies have reported greater mean and peak airflow in male than female singers, particularly at high pitches [6]. Hence, group differences in mean airflow in the current study might be explained, at least in part, by the predominance of females in the present group of classical singers. Although the current sample size does not allow sub-grouping based on gender, assessment of individual averages revealed that the male singers produced the highest mean airflow within the classical group (52 ± 25 mL/s). Two female singers demonstrated intermediate values (43 ± 22 mL/s) and the remaining two females demonstrated lower values (15 ± 11 mL/s). As the group average of the untrained individuals was substantially greater than all classical singers (114 ± 42 mL/s), gender effects cannot fully account for the group differences. Moreover, no significant differences were found in age, height, weight, or BMI between groups. Considering that two classical singers were more than 60 years old, it is important to consider that physiological age-related changes such as smaller vital capacities, reduced recoil of lung tissue, and reduced muscle strength may limit the range of vocal intensities in the elderly, potentially affecting vocal pitch, loudness, and quality [56]. The effects of these changes on the singing voice are highly variable, and depend on the level of vocal training. In non-singers, the fundamental frequency of voice is substantially lower in elderly compared to young adults, but not in elder professional singers [57], who are able to generate oral pressures that are similar to those of younger adults [58].

It is important to note the potential limitations of inductance plethysmography for recording respiratory kinematics. This device can only detect changes in the cross-sectional area of the cavities at the level of the bands and have limited capacity to detect distortion of rib-cage shape [59] or regional abdominal behaviour [60]. Despite this limitation, several methodological studies have demonstrated high accuracy of inductance plethysmography for the analysis of respiratory waveforms during quiet breathing and exercise [61,62].

## Conclusion

No significant differences were observed between the patterns of respiratory kinematics of untrained individuals and classical singers during quiet breathing, although there was a trend for greater abdominal contribution in singers than untrained individuals. In contrast, each group demonstrated distinct adaptations during singing tasks. Untrained individuals displayed minimal differences in the coordination between rib cage and abdominal movements with singing, which suggests similar respiratory patterns during both breathing and singing. Although there was some variation between participants, most classical singers demonstrated greater contribution of the abdomen to total lung volume than untrained individuals during singing tasks, and this was associated with pre-phonatory inward movements of the abdominal wall and a greater degree of independence in the movements of the rib cage and abdomen. This would increase the length and the pressure-generating capacity of rib cage expiratory muscles and elevate intra-abdominal pressure to improve the control of subglottal pressure during long utterances. These adaptations have been associated with changes in sound power spectrum and may have implications for voice quality, as commonly advocated by classical singing teachers and professionals.

## Supporting Information

S1 Dataset All parameters used in the data analysis, calculated for each individual during each task. (XLSX)
